# Study on Influencing Factors for Short-Term Symptom Resolution After Reinforced Radiculoplasty for Sacral Cysts: Focus on Bladder–Bowel Dysfunction

**DOI:** 10.3390/jcm15093196

**Published:** 2026-04-22

**Authors:** Wanzhong Yuan, Jiaxing Zhang, Hao Zhang, Weiwen Wang, Aoxue Mei, Jianjun Sun

**Affiliations:** Department of Neurosurgery, Beijing Friendship Hospital, Capital Medical University, Beijing 100050, China

**Keywords:** reinforced radiculoplasty, sacral cysts, bladder–bowel dysfunction, surgical management

## Abstract

**Background/Objectives**: Reinforced radiculoplasty (RRP) is effective for symptomatic sacral cysts, yet postoperative recovery varies significantly. This study aimed to systematically identify preoperative predictors of delayed short-term symptomatic recovery, with a specific focus on the prognostic impact of concomitant bladder–bowel dysfunction. **Methods**: A retrospective analysis was conducted on a cohort of 148 consecutive patients who underwent RRP. Comprehensive clinical, high-resolution imaging, and detailed surgical data were collected. The primary outcome was defined as symptom resolution within 9 months postoperatively. Independent prognostic factors were identified using univariate and subsequent multivariate logistic regression analysis. **Results**: Short-term symptom resolution was achieved in 86 patients (58.1%). Multivariate analysis identified three independent risk factors for prolonged recovery: a history of prior cyst surgery (OR 8.389, 95% CI 2.328–30.230), multi-segment cyst involvement (OR 2.682, 95% CI 1.066–6.744), and preoperative bladder–bowel dysfunction (OR 7.859, 95% CI 3.478–17.759). The predictive model demonstrated good discriminative ability (sensitivity 83.7%, specificity 61.3%). **Conclusions**: Prior surgical history, multi-segment cyst involvement, and preoperative bladder–bowel dysfunction are independent predictors of delayed short-term recovery after RRP for sacral cysts.

## 1. Introduction

Sacral cysts are extradural cystic lesions originating from spinal nerve root sleeves. Among them, Tarlov cysts are a characteristic subtype defined by nerve root fibers within the cyst wall [1]. While many cases are asymptomatic and discovered incidentally during imaging, cysts that progress and produce mass effects can cause persistent compression of the sacral nerve roots, leading to a range of clinical symptoms [2]. Notably, bladder and rectal dysfunction—such as voiding difficulty, urinary retention, or fecal incontinence—is particularly significant and can severely impair quality of life [3,4].

Surgical intervention remains the primary treatment for symptomatic sacral cysts [5,6,7,8,9,10]. Microscopic reinforced radiculoplasty (RRP), a nerve-preserving technique, has shown promise in relieving neural compression and reconstructing the nerve root sleeve [9,11]. However, postoperative recovery varies considerably among patients—some improve rapidly, while others experience slow or incomplete resolution, particularly of bladder and bowel symptoms. The timing of symptomatic recovery is clinically important but remains poorly understood. Specifically, which preoperative factors predict a delayed short-term (within 9 months) recovery is unknown. No previous study has systematically evaluated the prognostic value of preoperative bladder–bowel dysfunction, cyst morphology, multiplicity, or prior surgical history for the speed of symptom relief after RRP.

Therefore, this study aims to systematically investigate factors influencing the time to symptomatic relief following surgery through a retrospective analysis of 148 patients with sacral cysts who underwent RRP between August 2023 and June 2024. By comparing recovery outcomes among patients with different clinical manifestations, imaging characteristics, and surgical variables, and employing logistic regression to identify independent predictors of short- and long-term symptom improvement, this research seeks to identify patient subgroups at risk of prolonged recovery or requiring additional interventions. The findings are expected to inform preoperative evaluation, surgical strategy selection, and postoperative management, thereby facilitating the optimization of individualized treatment and rehabilitation approaches.

## 2. Materials and Methods

### 2.1. Study Sample

A total of 148 patients who underwent RRP between August 2023 and June 2024 were enrolled in this study. All patients underwent sacrococcygeal CT and high-resolution MRI within one week before surgery. Demographic and clinical characteristics were collected from medical records. The study protocol was approved by the Medical Science Research Ethics Committee. All patients signed written informed consent forms prior to participating in this study.

### 2.2. Research Methods

Preoperative examination: CT was performed to assess the sacral anatomical morphology and detect any sacral laminar defects. MRI with three-dimensional reconstruction was utilized to visualize the nerve roots within the sacral Tarlov cysts. After excluding patients with urinary system diseases and gastrointestinal functional disorders, urodynamic testing was performed to assess for neurogenic bladder dysfunction, and anal sphincter electromyography was conducted to evaluate for neurogenic bowel dysfunction.

MRI scans from all patients were reconstructed to examine spinal nerve roots within sacral Tarlov cysts. Imaging was performed using a 3.0 T sacral MRI system (GE Discovery MR750). Original axial, sagittal, and coronal T1- and T2-weighted images were processed on a GE Advantage Workstation 4.6 with 3D maximum intensity projection. Initial identification of nerve roots was conducted using axial T2-weighted images (T2WI), followed by multi-angle reconstruction of sagittal and coronal cyst views. Nerve roots demonstrating multiple directions or branching patterns were delineated in sagittal reconstructions. For each nerve and its branches, coronal images were generated in both parallel and perpendicular orientations. These reconstructed coronal images enabled detailed trajectory analysis of the nerve roots within the cysts, achieving comprehensive morphological reconstruction for analytical purposes.

Surgical operation: All procedures were performed by the same senior surgeon under microscopic guidance and continuous intraoperative neuroelectrophysiological monitoring. Under general anesthesia, the patient was placed in the prone position with a lumbosacral bridge. A customized midline incision was made over the sacral spinous processes; for reoperations, the incision was planned based on the previous scar. Bilateral sacral laminectomies were performed using an ultrasonic bone cutter to expose the terminal thecal sac.

Under microscopy, adhesions between the cyst wall, sacral nerve roots, and thecal sac were meticulously dissected, with careful identification and preservation of intraneural structures. Cerebrospinal fluid leakage sites were coagulated using low-power bipolar electrocautery (15 W) and reinforced with an artificial dural patch. For cysts < 1 cm in diameter, volume reduction was achieved by aspiration or low-power bipolar coagulation, followed by dural reconstruction. When no nerve roots were identified within the cyst, the fistula was suture-ligated. After cyst management, the surgical field was irrigated with saline, and the residual sacral canal cavity was filled with an absorbable gelatin sponge or a paravertebral muscle flap. Wound closure was performed in layers. Postoperatively, patients remained in the prone position for five days. For patients undergoing secondary surgery, due to the presence of variable degrees of scar adhesion and disrupted anatomical planes after the initial operation, more meticulous dissection of nerve roots from surrounding tissues is required to avoid neural injury. We routinely perform reinforced radiculoplasty combined with sacral neurolysis to relieve compression caused by recurrent cysts. For those with concomitant bladder–bowel dysfunction, a staged sacral neuromodulation (SNM) procedure is added: a temporary stimulation electrode is implanted in the first stage, and if satisfactory symptom improvement is achieved, a permanent pulse generator is implanted in the second stage. Further technical details are available in the Supplementary Materials, and a surgical video can be found in our previous report [12].

Postoperative complications and follow-up: Adverse events following surgery, including surgical site infection, cerebrospinal fluid leakage, and delayed wound healing, were systematically documented in this study. A phased imaging follow-up schedule was implemented, with radiological evaluations conducted at 3, 6, 9 and 12 months postoperatively, and annual assessments thereafter. Sacral magnetic resonance imaging (MRI) was performed to assess critical postoperative outcomes, such as sacral cyst recurrence and the presence of cerebrospinal fluid leakage. All enrolled patients completed a follow-up period of more than 1 year, during which the Japanese Orthopaedic Association (JOA) Lumbar Spine Score-related questionnaires were administered to comprehensively evaluate their postoperative functional recovery. The postoperative recurrent sacral cysts for patients were as follows: (1) Magnetic resonance imaging (MRI) results indicating the presence of sacral TCs; (2) The presence of clinical symptoms (such as severe lumbosacral pain, numbness, and claudication) and neurological deficits (including sphincter dysfunction and sexual dysfunction) associated with the sacral TCs; (3) A history of undergoing surgery for sacral TCs; (4) Within 3 months after the initial surgery for sacral TCs, either no relief of symptoms or a gradual aggravation, accompanied by MRI evidence of recurrence or an increase in the size of the original cysts; (5) Ineffectiveness of oral medication and a strong patient’s will for re-operation. The exclusion criteria were as follows: (1) Lack of correlation between clinical symptoms and anatomy; (2) Patients with concurrent lumbar pathology or insufficient clinical-radiological correlation; (3) Confusion of clinical symptoms with lumbar spinal stenosis or degenerative lumbar spine disease [12]. All postoperative sacral MRI scans (at 3, 6, 9, and 12 months, then annually) were independently reviewed by two experienced neuroradiologists blinded to clinical outcomes. Any disagreement was resolved by consensus. Short-term Symptom Resolution (within 9 months) was defined as achieving a JOA recovery rate of ≥50% at the 9-month postoperative follow-up, calculated as (postoperative JOA score − preoperative JOA score)/(29 − preoperative JOA score), combined with patient-reported main symptoms as “markedly improved” or “completely resolved”. Patients who did not meet these criteria (including all cases with a JOA recovery rate < 50%) were classified as Long-term Symptom Resolution. The 9-month postoperative endpoint was selected because: (1) in large animal models of long-distance nerve regeneration, electrophysiological recovery becomes detectable around 9 months [13]; and (2) clinical meta-analysis identifies 9 months as a critical cutoff for motor nerve repair, beyond which satisfactory outcomes decline markedly [14].

### 2.3. Statistical Analysis

Normally distributed continuous variables are expressed as the means and standard deviations, and nonnormally distributed variables are expressed as medians and interquartile ranges. Categorical variables are described as counts and percentages. The independent samples t-test, Mann–Whitney U test, chi-square test, and one-way analysis of variance (ANOVA) were used to compare baseline characteristics across patient groups stratified by the presence or absence of bowel/bladder dysfunction, by single versus multiple cysts, and by prognostic outcomes, and to assess differences in neurological recovery associated with different surgical approaches. The clinical features or cyst characteristics related to different neurological prognoses were analyzed as potential risk factors by using logistic regression analysis. Statistical analysis was performed using IBM SPSS Statistics 26.0 (SPSS Inc., Chicago, IL, USA). Statistical significance was determined as *p* < 0.05 (two-tailed).

Sample size estimation: Given the retrospective exploratory design of this study, sample size adequacy was assessed using the commonly accepted events-per-variable (EPV) criterion, which recommends a minimum of 10 outcome events per independent variable to obtain stable and reliable estimates in logistic regression models. Based on clinical experience and a systematic review of the literature, we planned to include approximately 5–6 core candidate predictors in the final multivariate model. Consequently, a minimum of 50–60 outcome events—defined as patients who did not achieve short-term symptom resolution within 9 months postoperatively—was required. Our study consecutively enrolled 148 patients, of whom 62 met the outcome definition. This provided an EPV > 10, exceeding the recommended threshold and ensuring the accuracy of the logistic regression analysis.

Handling of confounders: To control for potential confounding, we pre-specified age, sex, and symptom duration as a priori confounders based on clinical knowledge and directed acyclic graphs. These variables were forced into the final multivariable logistic regression model regardless of their statistical significance in univariate analysis. A hierarchical entry strategy was applied: Step 1 entered the a priori confounders; Step 2 added the remaining candidate predictors (those with *p* < 0.10 in univariate analysis and without significant multicollinearity). Collinearity was assessed using variance inflation factors (all VIFs < 2.5).

Justification for variable selection: Variables entered into the multivariable analysis included a priori core predictors (prior surgical history, multi-segment involvement, bladder–bowel dysfunction), a priori confounders (age, sex, symptom duration), and other variables with *p* < 0.10 in univariate analysis and no significant multicollinearity.

## 3. Results

### 3.1. Clinical Characteristics of the Study Population

A total of 148 patients were included, with 99 females and a mean age of 47.21 ± 13.81 years. The median preoperative symptom duration was 24 (9, 60) months. The primary clinical manifestations included lumbosacral and lower extremity symptoms in 126 patients (85.14%), perineal and perianal symptoms in 140 patients (94.6%), lower extremity motor dysfunction in 7 patients (4.73%), bladder dysfunction in 37 patients (25%), bowel dysfunction in 9 patients (6.08%), combined bladder–bowel dysfunction in 6 patients (4.05%), and history of previous sacral cyst surgery in 22 patients (14.86%).

Preoperative 3.0 T sacral MRI (GE Discovery MR750) was performed in all cases. The original images were transferred to a GE Advantage Workstation 4.6 and a 3D maximum intensity projection workstation for three-dimensional reconstruction to evaluate the spinal nerve roots within the sacral Tarlov cysts. Imaging characteristics revealed bilateral cysts in 83 patients (56.08%), Tarlov cysts in 132 patients (89.12%), single-segment involvement in 48 patients (32.43%), and sacral erosion in 93 patients (62.84%).

All patients underwent microsurgical RRP under neurophysiological monitoring. The mean operative time was 226.41 ± 147.03 min. Secondary revision surgery was performed in 22 patients (14.86%), consisting of revision RRP combined with sacral neurolysis in 14 patients (9.46%), RRP with Stage I sacral neuromodulation implantation in 4 patients (2.7%), and RRP with Stage II sacral neuromodulation implantation in 4 patients (2.7%).

All 148 enrolled patients completed the 9-month follow-up, and none were lost to follow-up, yielding a 100% follow-up rate for the primary outcome. During the postoperative follow-up period, no mortality or recurrence was documented. Significant symptom relief or resolution within 9 months was achieved in 86 patients (58.11%). Postoperative complications included surgical site fluid collection in 9 patients (6.08%) and delayed wound healing in 22 patients (14.86%). The detailed clinical characteristics are summarized in Table 1.

### 3.2. Comparison of Clinical Characteristics of Patients with and Without Bowel and Bladder Dysfunction

This study included 52 patients with bowel and bladder dysfunction and 96 patients without such dysfunction. Significant differences were observed between these two groups in terms of sex, duration of symptoms, lumbosacral and lower extremity symptoms, surgical history, number of cysts, polycystic patient, sagittal length, axial length, surgical approach, symptom resolution within 9 months, and delayed wound healing.

Notably, patients with sacral canal cysts accompanied by bowel and bladder dysfunction were more likely to be female. Furthermore, these patients showed a higher propensity to undergo Sacral Neurolysis and SNM Phase I. Patients with bowel and bladder dysfunction were less likely to experience symptom resolution within 9 months postoperatively, and a significant correlation was observed between the dysfunction and long-term recovery (Cramer’s V = 0.465, *p* < 0.001). The results are shown in Table 2.

**Table 1:** Baseline clinical characteristics of the study population.

	Patient Characteristics	Mean ± Standard Deviation, n (%)
**General Clinical Information**	**Age**, year	47.21 ± 13.81
	**Sex**, female	99 (66.90)
	**Hypertension**	10 (6.76)
	**Diabetes**	5 (3.38)
	**Coronary Disease**	3 (2.03)
	**Duration of Symptoms**, month	24, (9, 60)
	**Preoperative clinical symptoms**	
	Lumbosacral and Lower Extremity Symptoms	126 (85.14)
	Perineal and Perianal Symptoms	140 (94.60)
	Lower Extremity Motor Dysfunction	7 (4.73)
	Bladder and Bowel Dysfunction	52 (35.14)
	Bladder Dysfunction	37 (25.00)
	Bowel Dysfunction	9 (6.08)
	Both	6 (4.05)
	**Surgical History**	22 (14.86)
**Preoperative Imaging Examination and Cyst Characteristics**	**Preoperative JOA Score**	20, (18, 22)
	**Cyst Morphology**	
	Oval	60 (40.54)
	Beaded	38 (25.68)
	Irregular	50 (33.78)
	**Single-segment involvement**	48 (32.43)
	**Sacral Erosion**	93 (62.84)
	**Bilateral sacral**	83 (56.08)
	**Tarlov cysts**	132 (89.12)
	**Patients with multiple sacral cysts (≥3)**	55 (37.16)
	**Sagittal length**, mm	2.50 (1.62, 3.98)
	**Axial length**, mm	2.03 (1.33, 3.03)
**Intraoperative Clinical Information**	**Operation time**, minute	226.41 ± 147.03
	**Surgical manner**	
	Simple Microsurgical RRP	126 (85.14)
	Secondary Revision Surgery	22 (14.86)
	RRP and Sacral Neurolysis	14 (9.46)
	RRP with SNM Phase I	4 (2.70)
	RRP with SNM Phase II	4 (2.70)
**Post-operative Clinical Information**	**Symptom resolution (within 9 months)**	
	Short-term Symptom Resolution	86 (58.11)
	Long-term Symptom Resolution	62 (41.89)
	**Surgical Site Fluid Collection**	9 (6.08)
	**Delayed Wound Healing**	22 (14.86)

**Table 2:** Comparison of clinical characteristics of patients with and without bowel and bladder dysfunction.

KERRYPNX	Patient Characteristics	Mean ± Standard Deviation, n (%)		*p*-Value
		Bowel and Bladder Dysfunction (n = 52)	Without Bowel and Bladder Dysfunction (n = 96)	
**General Clinical Information**	**Age**, year	49.71 ± 13.84	45.85 ± 49.71	0.105
	**Sex**, female	41 (27.70)	58 (39.19)	0.023
	**Hypertension**	6 (4.05)	4 (2.70)	0.173
	**Diabetes**	0 (0.00)	1 (0.68)	0.351
	**Coronary Disease**	0 (0.00)	1 (0.68)	0.351
	**Duration of Symptoms**, month	45.46 ± 39.33	31.95 ± 30.89	0.046
	**Preoperative clinical symptoms**			
	Lumbosacral and Lower Extremity symptoms	89 (60.14)	37 (25.00)	<0.001
	Perineal and Perianal Symptoms	51 (34.46)	89 (60.14)	0.318
	Lower Extremity Motor Dysfunction	0 (0.00)	7 (4.73)	0.097
	**Surgical History**	12 (8.11)	10 (6.76)	0.039
**Preoperative Imaging Examination and Cyst Characteristics**	**Preoperative JOA Score**	18.62 ± 1.95	20.81 ± 1.59	<0.001
	**Cyst Morphology**			0.182
	Oval	18 (12.16)	42 (28.38)	
	Beaded	18 (12.16)	20 (13.51)	
	Irregular	16 (10.81)	34 (22.97)	
	**Single-segment involvement**	37 (25.00)	63 (42.57)	0.493
	**Sacral Erosion**	31 (20.95)	62 (41.89)	0.550
	**Bilateral sacral**	31 (20.95)	52 (35.14)	0.524
	**Number of cysts**	2.75 ± 1.75	2.14 ± 1.27	0.041
	**Tarlov cysts**	47 (31.76)	85 (57.43)	0.730
	**Patients with multiple sacral cysts (≥3)**	27 (18.24)	28 (18.92)	0.006
	**Sagittal length**, mm	2.61 ± 1.66	3.27 ± 2.00	0.027
	**Axial length**, mm	2.03 ± 1.21	2.42 ± 1.19	0.027
**Intraoperative Clinical Information**	**Operation time**, minute	118.31 ± 16.09	113.40 ± 13.92	0.054
	**Surgical manner**			0.005
	Simple Microsurgical RRP	38 (25.68)	86 (58.11)	
	Secondary Revision Surgery			
	RRP and Sacral Neurolysis	7 (4.73)	0 (0.00)	
	RRP with SNM Phase I	4 (2.70)	1 (0.68)	
	RRP with SNM Phase II	3 (2.03)	9 (6.08)	
**Post-operative Clinical Information**	**Symptom resolution (within 9 months)**			<0.001
	Short-term Symptom Resolution	14 (9.46)	72 (48.65)	
	Long-term Symptom Resolution	38 (25.68)	24 (16.22)	
	**Surgical Site Fluid Collection**	2 (1.35)	7 (4.73)	0.633
	**Delayed Wound Healing**	12 (8.11)	10 (6.76)	0.039

### 3.3. Comparison of Clinical Characteristics of Patients with and Without Polycystic (Number of Cysts ≥ 3)

This study included 55 polycystic patients. Significant differences were observed between these two groups in terms of lumbosacral and lower extremity symptoms, cyst morphology, single-segment involvement, sacral erosion, Tarlov cysts, bilateral sacral, bowel and bladder dysfunction, sagittal length, axial length, operation time and symptom resolution within 9 months.

Patients with multiple sacral cysts (≥3) typically exhibit beaded, bilateral cysts that involve multiple sacral segments and are associated with bone erosion. Clinically, they most commonly present with lumbosacral pain and bowel/bladder dysfunction, which are less likely to resolve rapidly within 9 months post-surgery. The results are shown in Table 3.

### 3.4. Comparison of Clinical Characteristics of Patients with Different Symptom Resolution

This study included 86 patients with short-term symptom resolution (within 9 months) and 62 patients with long-term symptom resolution. Significant differences were observed between these two groups in terms of surgical history, cyst morphology, multiple sacral cysts (≥3), bowel and bladder dysfunction, operation time, surgical manner and delayed wound healing.

Patients who achieved long-term postoperative symptom resolution were more likely to have beaded cysts, a history of prior surgery, and concomitant bowel/bladder dysfunction. The results are shown in Table 4.

### 3.5. Association of Clinical Related Factors with Different Symptom Resolution

The results of the regression analysis are shown in Table 5. Univariate logistic regression analysis showed that surgical history, preoperative JOA Score, beaded cyst, multiple sacral cysts, bowel/bladder dysfunction and operation time were correlated with long-term symptom resolution.

After correcting for confounders, multifactorial logistic regression analysis showed that surgical history (OR, 8.389; 95% CI, 2.328–30.230; *p* = 0.001), multi-segment involvement (OR, 2.682; 95% CI, 1.066–6.744; *p* = 0.036) and bowel and bladder dysfunction (OR, 7.859; 95% CI, 3.478–17.759; *p* < 0.001) were correlated with different symptom resolution

The logistic regression model was statistically significant (Omnibus Test of Model Coefficients, *p* < 0.001) and correctly classified 74.3% of the cases. Hosmer–Lemeshow test (*p* = 0.785). The model demonstrated a sensitivity of 83.7%, a specificity of 61.3%, a positive predictive value (PPV) of 75.79%, and a negative predictive value (NPV) of 73.77%.

**Table 3:** Comparison of clinical characteristics of patients with and without multiple sacral cysts (Number of cysts ≥ 3).

	Patient Characteristics	Mean ± Standard Deviation, n (%)		*p*-Value
		Without Polycystic (n = 93)	Polycystic (n = 55)	
**General Clinical Information**	**Age**, year	46.23 ± 14.22	48.87 ± 13.04	0.261
	**Sex**, female	62 (41.89)	37 (25.00)	0.940
	**Hypertension**	4 (2.70)	6 (4.05)	0.122
	**Diabetes**	0 (0.00)	1 (0.68)	0.192
	**Coronary Disease**	0 (0.00)	1 (0.68)	0.192
	**Duration of Symptoms**, month	38.57 ± 36.36	33.53 ± 31.38	0.616
	**Preoperative clinical symptoms**			
	Lumbosacral and Lower Extremity symptoms	86 (58.11)	40 (27.03)	0.001
	Perineal and Perianal Symptoms	88 (59.46)	52 (35.14)	0.984
	Lower Extremity Motor Dysfunction	5 (3.38)	2 (1.35)	0.630
	**Surgical History**	93 (62.84)	55 (37.16)	0.383
**Preoperative Imaging Examination and Cyst Characteristics**	**Preoperative JOA Score**	20.33 ± 1.75	19.55 ± 2.32	0.014
	**Cyst Morphology**			<0.001
	Oval	49 (3311)	11 (7.43)	
	Beaded	7 (4.73)	31 (20.95)	
	Irregular	37 (25.00)	13 (8.78)	
	**Single-segment involvement**	38 (25.68)	10 (6.76)	0.004
	**Sacral Erosion**	64 (43.24)	29 (19.59)	0.050
	**Bilateral sacral**	31 (20.95)	52 (35.14)	<0.001
	**Tarlov cysts**	77 (52.03)	55 (37.16)	0.001
	**Bowel and bladder dysfunction**	25 (16.89)	27 (18.24)	0.006
	**Sagittal length**, mm	3.44 ± 2.13	2.35 ± 1.18	0.001
	**Axial length**, mm	2.50 ± 1.24	1.91 ± 1.07	0.004
**Intraoperative Clinical Information**	**Operation time**, minute	106.71 ± 9.59	129.35 ± 10.76	<0.001
	**Surgical manner**			0.425
	Simple Microsurgical RRP	80 (54.05)	44 (29.73)	
	Secondary Revision Surgery			
	RRP and Sacral Neurolysis	10 (6.76)	6 (4.05)	
	RRP with SNM Phase I	1 (0.68)	3 (2.03)	
	RRP with SNM Phase II	2 (1.35)	2 (1.35)	
**Post-operative Clinical Information**	**Symptom resolution (within 9 months)**			0.002
	Short-term Symptom Resolution	63 (42.57)	23 (15.54)	
	Long-term Symptom Resolution	30 (20.27)	32 (21.62)	
	**Surgical Site Fluid Collection**	4 (2.70)	5 (3.38)	0.239
	**Delayed Wound Healing**	12 (8.11)	10 (6.76)	0.383

**Table 4:** Comparison of clinical characteristics of patients with different symptom resolution.

KERRYPNX	Patient Characteristics	Mean ± Standard Deviation, n (%)		*p*-Value
		Long-Term Symptom Resolution (n = 62)	Short-Term Symptom Resolution (Within 9 Months) (n = 86)	
**General Clinical Information**	**Age**, year	48.24 ± 13.57	46.47 ± 14.01	0.442
	**Sex**, female	45 (30.41)	54 (36.49)	0.212
	**Hypertension**	5 (3.38)	5 (3.38)	0.590
	**Diabetes**	0 (0.00)	1 (0.68)	0.394
	**Coronary Disease**	1 (0.68)	0 (0.00)	0.237
	**Duration of Symptoms**, month	42.79 ± 38.49	32.30 ± 30.94	0.136
	**Preoperative clinical symptoms**			
	Lumbosacral and Lower Extremity symptoms	49 (33.11)	77 (52.03)	0.076
	Perineal and Perianal Symptoms	60 (40.54)	80 (54.05)	0.319
	Lower Extremity Motor Dysfunction	1 (0.68)	6 (4.05)	0.129
	**Surgical History**	17 (11.49)	5 (3.38)	<0.001
**Preoperative Imaging Examination and Cyst Characteristics**	**Preoperative JOA Score**	19.39 ± 2.11	20.51 ± 1.81	0.134
	**Cyst Morphology**			0.007
	Oval	17 (11.49)	43 (29.05)	
	Beaded	23 (15.54)	15 (10.14)	
	Irregular	22 (14.86)	28 (18.92)	
	**Single-segment involvement**	16 (10.81)	32 (21.62)	0.144
	**Sacral Erosion**	38 (25.68)	55 (37.16)	0.741
	**Bilateral sacral**	37 (25.00)	46 (31.08)	0.454
	**Tarlov cysts**	56 (37.84)	76 (51.35)	0.706
	**Multiple sacral cysts (≥3)**	32 (21.62)	23 (15.54)	0.002
	**Bowel and bladder dysfunction**	38 (25.68)	14 (9.46)	<0.001
	**Sagittal length**, mm	2.86 ± 1.75	3.16 ± 2.01	0.304
	**Axial length**, mm	2.09 ± 1.75	2.42 ± 1.25	0.087
**Intraoperative Clinical Information**	**Operation time**, minute	119.34 ± 16.99	112.08 ± 12.34	0.001
	**Surgical manner**			<0.001
	Simple Microsurgical RRP	43 (29.05)	81 (54.73)	
	Secondary Revision Surgery			
	RRP and Sacral Neurolysis	11 (7.43)	0 (0.00)	
	RRP with SNM Phase I	4 (2.70)	0 (0.00)	
	RRP with SNM Phase II	4 (2.70)	5 (3.38)	
**Post-operative Clinical Information**	**Surgical Site Fluid Collection**	6 (4.05)	3 (2.03)	0.228
	**Delayed Wound Healing**	17 (44.49)	5 (3.38)	<0.001

**Table 5:** Univariate and multivariate logistic regression analysis of factors associated with symptom resolution.

	Patient Characteristics	Univariate Regression			Multivariate Regression Model		
		OR	Beta (95% CI)	*p*-Value	OR	Beta (95% CI)	*p*-Value
**General Clinical Information**	**Age**	0.991	−0.009 (0.967–1.015)	0.439			
	**Sex** reference: female	0.638	−0.450 (0.314–1.295)	0.213			
	**Duration of Symptoms**	0.991	−0.009 (0.982–1.001)	0.071			
	**Preoperative clinical symptoms**						
	Lumbosacral and Lower Extremity symptoms	0.441	−0.820 (0.175–1.108)	0.082			
	Perineal and Perianal Symptoms	2.250	0.811 (0.439–11.541)	0.331			
	Lower Extremity Motor Dysfunction	0.219	−1.521 (0.026–1.863)	0.164			
	**Surgical History** reference: without surgical history	6.120	1.812 (2.117–17.693)	0.001	8.389	2.127 (2.328–30.230)	0.001
**Preoperative Imaging Examination and Tumor Characteristics**	**Preoperative JOA Score**	1.351	0.301 (1.126–1.620)	0.001			
	**Cyst Morphology**						
	Beaded	0.258	−1.355 (0.109–0.609)	0.002			
	Irregular	0.503	−0.687 (0.228–1.111)	0.089			
	reference: oval						
	**Multi-segment involvement** reference: single-segment involvement	0.587	−0.533 (0.286–1.203)	0.146	2.682	0.986 (1.066–6.744)	0.036
	**Sacral Erosion**	0.892	−0.114 (0.455–1.752)	0.741			
	**Bilateral sacral**	1.287	0.252 (0.664–2.493)	0.454			
	**Tarlov cysts**	1.228	0.205 (0.422–3.578)	0.707			
	**Multiple sacral cysts (≥3)**	2.922	1.072 (1.465–5.825)	0.002			
	**Bowel and bladder dysfunction**	8.143	2.097 (3.780–17.541)	<0.001	7.859	2.062 (3.478–17.759)	<0.001
	**Sagittal length**, mm	1.089	0.085 (0.911–1.301)	0.348			
	**Axial length**, mm	1.270	0.239 (0.956–1.686)	0.099			
**Intraoperative Clinical Information**	**Operation time**, minute	0.966	−0.034 (0.944–0.989)	0.004			
	**Surgical manner**						
	Secondary Revision Surgery						
	RRP and Sacral Neurolysis	0.100	−21.836 (0.012–17.450)	0.999			
	RRP with SNM Phase I	0.100	−21.836 (0.012–17.450)	0.999			
	RRP with SNM Phase II	0.241	−1.422 (0.079–0.074)	0.013			
	reference: Simple Microsurgical RRP						

## 4. Discussion

Reinforced radiculoplasty (RRP) has become an effective surgical procedure for symptomatic sacral cysts, particularly Tarlov cysts [12,15,16]. Through a systematic analysis of 148 patients undergoing RRP, this study comprehensively identified key factors influencing short-term symptomatic recovery. Multivariate logistic regression revealed that prior surgical history, multi-segment cyst involvement, and preoperative bowel/bladder dysfunction were independent risk factors impeding short-term symptom relief. These findings provide valuable insights for preoperative risk assessment and personalized surgical planning.

The identification of surgical history as an independent risk factor carries significant clinical implications. This study demonstrated that patients with a prior surgical history had a significantly reduced likelihood of achieving short-term symptomatic relief (OR = 8.389). This finding prompts a deeper consideration of the technical challenges inherent to reoperation. Beyond issues like post-operative scarring and disrupted anatomical planes, we propose that the more critical factor may be the irreversible alteration of the neural interface microenvironment. Primary surgical techniques, such as fat/muscle packing or the use of biological glues, may not only cause renewed irritation to exposed nerve roots but also disrupt the nutritional exchange balance between the nerve roots and surrounding tissues [12]. It is possible that previous surgery alters the perineural environment, which may affect neural recovery, although this hypothesis requires further investigation [17]. Clinically, even with meticulous dissection during reoperation, functional recovery of the nerve often remains suboptimal. This underscores the importance of employing more refined techniques during the initial surgery, focusing on reconstructing the anatomical integrity of the nerve roots while minimizing disturbance to the perineural environment. Additionally, the intraoperative application of anti-adhesion materials should be considered to preserve options for potential future interventions. In practice, the RRP procedure has shown favorable results in addressing these aspects.

The clinical implications of multi-segment cysts for surgical outcomes should be further understood from the perspective of neural innervation. Our results indicate that multi-segment involvement is associated with a “beaded” cyst morphology (*p* < 0.001) and sacral erosion (*p* = 0.050). Such complex anatomical characteristics pose greater surgical challenges. Intraoperatively, we have observed that multi-segment sacral cysts often exhibit an overlapping functional zones phenomenon, where neurophysiological monitoring suggests that nerves within a single cyst can simultaneously affect multiple neural functional units and their corresponding muscle innervation. This necessitates that surgeons focus not only on cyst decompression but also on functional reconstruction. We recommend adopting a sequential surgical strategy of “neuromapping → function reconstruction.” This involves precise intraoperative neurophysiological mapping to identify critical functional areas, prioritizing their protection before pursuing maximal cyst resection. Although this approach may extend operative time, it is essential for preserving neurological function and facilitating postoperative recovery.

The predictive value of bowel and bladder dysfunction should be interpreted within the overall functional assessment of the pelvic floor neural network. This study confirmed that such dysfunction is significantly associated with female sex, longer symptom duration, and multiple cysts. From a neuroanatomical perspective, bladder and rectal functions are finely regulated by the pelvic plexus, primarily composed of the S2–S4 nerve roots [18,19]. Under chronic compression from cysts, these nerves are susceptible to neurapraxia. In patients undergoing reoperation, more severe injuries—such as axonotmesis or even neurotmesis—may occur following the initial surgery, potentially leading to varying degrees of bowel and bladder dysfunction [20]. While mild nerve impairment often recovers well after decompression, severe damage results in limited functional improvement even after successful surgery [21]. This classification holds important clinical implications: for neurapraxia, RRP alone may suffice, whereas more severe nerve injuries may require a combined approach of RRP and sacral neuromodulation (SNM) [22]. We recommend preoperative evaluation of nerve injury severity through urodynamic studies, anal sphincter electromyography, and high-resolution MRI to guide surgical planning. Notably, the combined strategy of RRP and SNM offers a promising therapeutic alternative for severe cases. The synergistic mechanism may involve RRP alleviating mechanical compression, while SNM promotes neural functional remodeling through electrical stimulation.

Based on these findings, we propose an individualized treatment strategy guided by risk stratification. Surgical management should be tailored according to patient risk levels as follows:

Low-risk patients (primary surgery, short symptom duration, single-segment involvement, no bowel/bladder dysfunction) may be considered for standard RRP. Intermediate-risk patients (multi-segment or multiple cysts, prolonged symptom duration, prior surgical history) may require enhanced neural protection during RRP, such as adjuvant neurotrophic medication postoperatively. High-risk patients (with preoperative bowel/bladder dysfunction) may benefit from a combined RRP and sacral neuromodulation (SNM) approach. However, this suggestion is hypothesis-generating given the limited number of SNM recipients in our cohort (n = 8) and warrants prospective investigation. This stratified treatment strategy aims to ensure therapeutic efficacy while minimizing the risks associated with overtreatment.

This study was conducted at a single institution with all procedures performed by one experienced surgeon. While this minimizes technical variability, the findings require external validation in multicenter cohorts. The retrospective nature of this study may introduce selection bias despite consecutive enrollment and covariate adjustment. Prospective studies are warranted to confirm our findings. The lack of validated questionnaires for assessing bladder–bowel dysfunction is a limitation of this retrospective study, which will be addressed in future prospective studies.

## 5. Conclusions

This study identifies that prior surgical history, multi-segment sacral cyst involvement, and preoperative bowel/bladder dysfunction are independent risk factors for impaired short-term recovery following reinforced radiculoplasty (RRP) for sacral cysts. Given the retrospective design, these findings require further validation in prospective cohorts.

## Data Availability

The original contributions presented in this study are included in the article/Supplementary Material. Further inquiries can be directed to the corresponding author.

## Supplementary Materials

The following supporting information can be downloaded at: https://www.mdpi.com/article/10.3390/jcm15093196/s1, File S1: Surgical operation.

## Abbreviations

The following abbreviations are used in this manuscript:

RRP	Reinforced radiculoplasty
T2WI	T2-weighted images
SNM	Sacral neuromodulation
MRI	Magnetic resonance imaging
JOA	Japanese Orthopaedic Association
