# Supplementary material for: Study on Influencing Factors for Short-Term Symptom Resolution After Reinforced Radiculoplasty for Sacral Cysts: Focus on Bladder–Bowel Dysfunction
Source: J Clin Med. 2026 Apr 22;15(9):3196. doi: 10.3390/jcm15093196 (PMC13164424; doi:10.3390/jcm15093196)
Supplement: Supplementary file 1 [file jcm-15-03196-s001.zip › jcm-4200606-supplementary.pdf]

File S1

Surgical operation:

All surgical procedures were performed under microscopic guidance by the same chief surgeon. Under general anesthesia, patients were placed in a prone position using a lumbosacral bridge support. Cyst exploration combined with RRP was performed, with some patients undergoing reoperation also receiving concurrent sacral sacral neuromodulation (SNM). Continuous intraoperative neuroelectrophysiological monitoring was maintained throughout the procedure.

A customized midline incision was made over the sacral spinous processes. For patients undergoing reoperation, the incision was planned based on the location of the recurrent cysts relative to the previous surgical scar. Sequential dissection was carried down to the paraspinal muscles, which were mobilized to expose the posterior sacral wall. Bilateral sacral laminectomies were performed using an ultrasonic bone cutter to expose the terminal portion of the thecal sac.

Under microscopic visualization, adhesions between the cyst wall, sacral nerve roots, and thecal sac terminus were carefully dissected and released. Meticulous attention was directed to identifying and preserving intraneural structures, delineating nerve root entry and exit zones, and confirming the absence of neural attachments to the cyst wall proper.

Cerebrospinal fluid leakage sites were coagulated and narrowed using low-power bipolar electrocautery (15 W) and reinforced with an artificial dural patch. For cysts smaller than 1 cm in diameter, volume reduction was achieved either by aspiration or low-power bipolar coagulation, followed by reconstruction and reinforcement using an artificial dural substitute. In cases where no nerve roots were present within the cyst, the cyst wall was dissected, the fistula identified, and subsequently suture-ligated.

Following cyst management, the surgical field was irrigated with saline with the patient's head elevated to dynamically assess for leakage and ensure non-distention of the nerve root sleeves. The residual sacral canal cavity was filled with an absorbable gelatin sponge or a mobilized paravertebral muscle flap. Formal reconstruction was performed if the posterior sacral wall

remained intact. Wound closure was completed in layers. Postoperatively, patients maintained a prone position for five days.

For patients undergoing secondary surgery without bowel or bladder dysfunction, RRP of the recurrent cyst combined with sacral neurolysis is performed. For those with concomitant bowel and bladder dysfunction, SNM is additionally considered in conjunction with the aforementioned procedure.

During the procedure, after addressing the cyst, the SCL302C sacral nerve stimulation electrode is then inserted through the S3 sacral foramen. Under fluoroscopic guidance, the electrode is precisely positioned along the target nerve pathway. A temporary stimulator is connected, impedance is confirmed to be satisfactory, and the stimulation points are verified to accurately cover the innervation area of the target nerve roots. Finally, the stimulator lead is secured subcutaneously below the ipsilateral posterior superior iliac spine.

Postoperatively, an external stimulator is connected, and stimulation parameters and frequency are dynamically adjusted based on the patient's subjective feedback. If the patient reports significant improvement in bowel and bladder dysfunction compared to preoperative status during the trial period, a second-stage SNM procedure is performed: under local anesthesia, a 3 cm incision is made above the lateral aspect of the buttock. The electrode tail is tunneled subcutaneously, connected to the permanent pulse generator (SCS301B, from Rishena Medical Co., Ltd.), and the entire system is implanted subcutaneously before the incision is closed in layers. Follow-up is conducted monthly postoperatively, with further fine-tuning of parameters based on symptomatic response
